# Electrophysiological Correlates of Changes in Reaction Time Based on Stimulus Intensity

**DOI:** 10.1371/journal.pone.0036407

**Published:** 2012-05-03

**Authors:** Bimal Lakhani, Albert H. Vette, Avril Mansfield, Veronica Miyasike-daSilva, William E. McIlroy

**Affiliations:** 1 Graduate Department of Rehabilitation Science, University of Toronto, Toronto, Ontario, Canada; 2 Mobility Research Team, Toronto Rehab, Toronto, Ontario, Canada; 3 Department of Kinesiology, University of Waterloo, Waterloo, Ontario, Canada; Tokyo Metropolitan Institute of Medical Science, Japan

## Abstract

**Background:**

Although reaction time is commonly used as an indicator of central nervous system integrity, little is currently understood about the mechanisms that determine processing time. In the current study, we are interested in determining the differences in electrophysiological events associated with significant changes in reaction time that could be elicited by changes in stimulus intensity. The primary objective is to assess the effect of increasing stimulus intensity on the latency and amplitude of afferent inputs to the somatosensory cortex, and their relation to reaction time.

**Methods:**

Median nerve stimulation was applied to the non-dominant hand of 12 healthy young adults at two different stimulus intensities (HIGH & LOW). Participants were asked to either press a button as fast as possible with their dominant hand or remain quiet following the stimulus. Electroencephalography was used to measure somatosensory evoked potentials (SEPs) and event related potentials (ERPs). Electromyography from the flexor digitorum superficialis of the button-pressing hand was used to assess reaction time. Response time was the time of button press.

**Results:**

Reaction time and response time were significantly shorter following the HIGH intensity stimulus compared to the LOW intensity stimulus. There were no differences in SEP (N20 & P24) peak latencies and peak-to-peak amplitude for the two stimulus intensities. ERPs, locked to response time, demonstrated a significantly larger pre-movement negativity to positivity following the HIGH intensity stimulus over the Cz electrode.

**Discussion:**

This work demonstrates that rapid reaction times are not attributable to the latency of afferent processing from the stimulated site to the somatosensory cortex, and those latency reductions occur further along the sensorimotor transformation pathway. Evidence from ERPs indicates that frontal planning areas such as the supplementary motor area may play a role in transforming the elevated sensory volley from the somatosensory cortex into a more rapid motor response.

## Introduction

For well over a century, reaction time has been utilized as a window into the functionality of the central nervous system (CNS) [1], [2]. This interest has been based on the idea that the time to initiate a response reveals important insight into the pathways, processing and overall health of the CNS [3], [4]. As a result, there is an immense body of literature that has explored the factors that influence reaction time. These include, but are not limited to, age [5], [6], gender [7], [8], anticipation [9], stimulus modality [1], [9], arousal [10], task urgency [11], [12], [13] and stimulus intensity [14], [15]. While there has been tremendous effort to identify the factors that may influence reaction time, there has been far less on the underlying CNS substrates that may influence the time to process stimuli. The implications of such work would effect the interpretation of performance or health related changes in reaction time and influence possible strategies to improve time of processing when impaired by neurologic injury.

Factors that influence reaction time can be broken down into two main categories: 1) **characteristics** of the network and 2) **modulators** of the network. The first, and more obvious, are the characteristics of the network sub-serving the stimulus response transformation, including axon length, conduction velocity, and number/type of intervening synapses. Differences in network characteristics can account, in part, for differences between task conditions (such as simple versus choice reactions or monosynaptic versus polysynaptic reflexes). The second factor, modulators, can influence the reaction time of a specific task relying on the *same* network. These modulators can include anticipation, attention, arousal, and stimulus intensity. However, the underlying mechanisms of these modulators are not well understood and may serve to be the foundation for the most rapid reactions, such as ‘temporally-urgent’ reactions, which protect the individual against harm [11], [16] and which are often compromised after neurologic injury [4], [17]. The purpose of the present work is to provide a fundamental understanding of the cortical events associated with a reduction of reaction time that is associated with a modulator – specifically, stimulus intensity. Although increased stimulus intensity has been cited as a cause for reduced reaction times [15], [18], [19], the CNS mechanism that allows for dramatic reductions, such as those following a temporally urgent stimulus (∼150 ms) [12], remains unclear.

The present study is focused on the sensory and motor-related activity at the level of the cortex during a rapid simple reaction time task. Specifically, the study is designed to determine whether the properties of the sensory or motor cortical events relate to reductions in reaction time that are induced by increases in stimulus intensity. In order to reveal discrete sensory events, the stimulus used in this paradigm is a direct electrical stimulation of a peripheral nerve. The resulting somatosensory evoked potentials (SEPs) following median and ulnar nerve stimulation in humans have been well described [20], [21], [22], [23]. These studies demonstrated notable activity in the central and parietal areas of the cortex contralateral to the stimulated nerve, which manifested with six early cortical potentials (P10, P12, P14, N19, P20 and P23). These short latency SEPs reflect the timing of sensory propagation through the brachial plexus and eventually into the somatosensory cortex [23]. However, the fastest conducting sensory fibers have a lower threshold voltage than slower fibers, suggesting that recruitment would have little effect on conduction latency [24]. More recently, Legon et al. [25] demonstrated that the frontal N30 component is activated during the execution, but not the preparation, of a movement contralateral to the site of electrical stimulation, signifying an important link between early sensory and motor cortical components of movement. The existence of early cortical SEPs that are independent of movement preparation, but specifically tied to movement execution, provides an opportunity to explore the contribution of afferent inputs to the reduction of reaction time latencies following a high intensity stimulus. The foremost of these questions being: are early SEPs, such as the N20 and P24, susceptible to variations in amplitude and latency based on increased stimulus intensity and how does the inclusion of a motor task affect the amplitudes and latencies of these SEPs compared to a purely sensory task?

Of additional importance to this study are long latency event related potentials (ERPs) that peak approximately 150–400 ms following the presentation of a stimulus. ERPs recorded from the surface of the scalp provide good temporal resolution and assist in the determination of the course of motor and cognitive events. A large negativity is generated approximately 250 ms following somatosensory stimulus presentation (N250) and is thought to reflect attentive processes, which are temporally linked to a behavioral response [26]. Subsequent to the N250, a large positivity occurs approximately 300 ms following stimulus presentation (P300). The P300 is thought to be associated with working memory stores, and its amplitude is proportional to the amount of attentional resources given to a task [27], [28]. Although these ERPs have been demonstrated previously, there is still a limited understanding regarding the relation between stimulus intensity, ERP amplitude/latency characteristics, and response time.

In the current study, we are interested in understanding the effects of stimulus intensity on reaction time and associated differences in the underlying electro-cortical responses. In other words, where along the cortical transformation pathway could one account for the potential stimulus dependent changes in reaction time? Importantly, if stimulus intensity has an effect on response latency as expected [14], [18], we are specifically interested in the potential differences in timing and amplitude of electro-cortical events. Such investigation of the timing and amplitude of early SEPs would provide evidence regarding the contribution of input (stimulus reception) to reducing the latency of reaction times. Conversely, if the temporal profile of SEPs remains unchanged despite shorter reaction times, it raises the possibility that reduction of timing arises from later phases of the sensorimotor transformation such as a more rapid cortical integration or even efferent conduction time. The motor cortical events will be explored by comparing task related differences in ERPs (time-locked to the responses). To the best of our knowledge, this is the first study that seeks to determine the possible source for changes in reaction time and that relies both on SEP and ERP approaches to disentangle sensory and motor contributions. We view that such fundamental work is important to advance our understanding of the determinants of speed of processing within the central nervous system.

## Methods

### Ethics Statement

This study has been reviewed and approved by the Office of Research Ethics at the University of Waterloo (as well as the Research Ethics Board at the Toronto Rehabilitation Institute). All participants provided informed, written consent.

### Participants

Twelve healthy, right-hand dominant, young adults participated in this study. The mean age of the participants was 26±6 years. None of the participants had any neurologic or musculoskeletal disorders that may have affected the ability to complete the tasks.

### Behavioral Task

**Figure 1:**
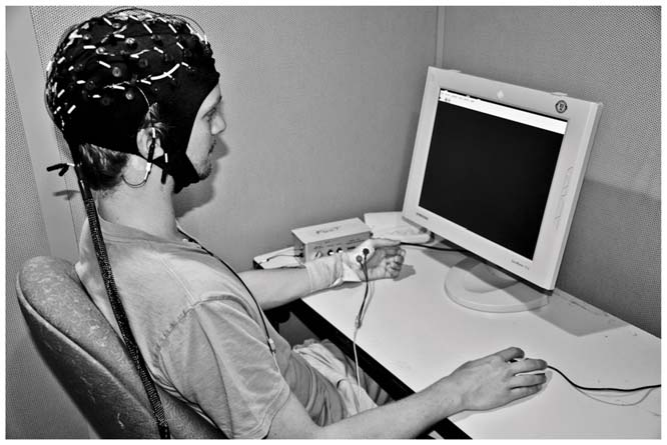
Overall layout of participant setup. Participants were seated in a sound-proof booth received median nerve stimulations to the non-dominant hand and either a HIGH or LOW stimulus intensity.

Participants were seated comfortably in a chair with both arms resting on a table in front of them in a sound-isolated booth (**Figure 1**). Two task conditions were completed in blocks following the presentation of a temporally unpredictable single-pulse transcutaneous electrical stimulus. In the MOTOR task, participants were asked to respond as quickly as possible by pressing a mouse button with their dominant index finger, whereas in the SENSORY task, participants were instructed not to react to the electrical stimulus. Prior to each stimulus delivery, participants were cued with a visual signal presented on an LED screen located approximately 30 cm in front of the participant. The electrical stimulus followed the visual cue at a random time 2 to 7 seconds later. Task conditions (MOTOR/SENSORY) were delivered in alternating blocks of 20 trials, while stimulus intensity was randomized within the blocks between two possible intensities (HIGH/LOW). A total of 200 trials were completed (50 per combination of task (MOTOR/SENSORY) and intensity (HIGH/LOW). Rest breaks were scheduled after each block of 20 trials.

### Stimulation & Recording

Electrical stimuli consisted of 1 ms square waves delivered through a surface bar electrode, with the anode distal, placed over the median nerve of the non-dominant upper limb at the wrist (GRASS S88 stimulator with SIU5 stimulus isolation unit; West Warwick, Rhode Island, USA). Motor threshold (MT) intensity was determined by gradually increasing stimulus intensity until a slight twitch could be visually noticed in the thenar eminence on the stimulated side. Two stimulus intensities were used: the LOW stimulus intensity was set at 0.8×MT, while the HIGH intensity was set to 1.5×MT. Response time was defined by the time of mouse click in the MOTOR condition relative to stimulus delivery time using a custom LabView program (National Instruments; Austin, Texas, USA). Electroencephalographic (EEG) data were recorded from 11 electrode sites (FCz, Cz, CPz, C1, C2, C3, C4, CP1, CP2, CP3, CP4), in accordance with the international 10–20 system for electrode placement referenced to the linked mastoids (impedance <5 kΩ). EEG data were amplified (40000×), filtered (DC-200 Hz), digitized at 1000 Hz (NeuroScan 4.3; Compumedics; El Paso, Texas, USA), and stored on a computer for offline analysis.

Surface electromyography (EMG) was recorded from the flexor digitorum superficialis (FDS) of the dominant upper limb. EMG was also recorded from the thenar musculature of the non-dominant hand to record M-wave activity following the electrical stimulus. M-wave peak-to-peak onset latency and amplitude were measured to confirm the consistency of the electrical stimulus intensity. EMG electrode sites were cleaned with alcohol and abrasive cream, and shaved if necessary. Silver/silver chloride electrodes were fixed 1 cm apart over the muscle belly. EMG signals were amplified at a gain of 2000× and stored for offline processing, using customized Lab View software (National Instruments; Austin, Texas, USA).

### Data Analysis

EMG signals were digitally filtered from 20 to 500 Hz (2^nd^ order zero phase-lag Butterworth) and conditioned by removing any DC offset bias and by full-wave rectifying the signal. Reaction time was determined from the EMG onset latency as the time following stimulus delivery when the EMG amplitude exceeded five standard deviations from the mean of a 100 ms baseline value taken prior to the stimulus delivery. EMG amplitude was calculated as the total integrated EMG activity (iEMG) for 100 ms following EMG onset. iEMG amplitude was normalized relative to each subject's mean iEMG in the HIGH intensity/MOTOR condition.

SEPs were measured from individual participant averages of all response epochs from the electrode site that displayed maximal activity (CP4). SEPs were extracted by averaging epochs time-locked to median nerve stimulation (−100 to 100 ms). Latencies were measured from the stimulus onset to the peak of each SEP (parietal N20 and P27). The N20–P27 amplitude was measured as the peak-to-peak amplitude between the two SEPs. ERPs were extracted by averaging epochs time-locked to the response time. The amplitude and latency of distinct ERPs prior to response time were assessed from all recorded EEG sites. Response time was used for ERPs due to the consistent nature of the square wave pulse that was evoked following the button press, whereas the precise onset of EMG activity from FDS can be difficult to detect and inconsistent. Individual SEP and ERP traces were high-pass filtered (2 Hz) and visually inspected for artifacts. A clearly defined peak was necessary for inclusion. Any contaminated epochs were eliminated before averaging.

### Statistics

Response times (mouse click), reaction times (FDS EMG onset) and iEMG amplitudes in the MOTOR task were analyzed using one-way ANOVAs with stimulus intensity (HIGH/LOW) as a repeated factor. M-wave onset latency in the HIGH stimulus conditions was analyzed by a one-way ANOVA with task condition (MOTOR/SENSORY) as a repeated factor. SEP onset latency and amplitude were analyzed using a two-way ANOVA with task condition and stimulus intensity as repeated factors. Regarding ERPs, peak-to-peak amplitude of the pre-movement negativity (PreN) to pre-movement positivity (PreP) as well as the onset latency of these peaks, in the MOTOR task condition was analyzed using a one-way ANOVA (HIGH vs. LOW). Significance levels were set at p<0.05. Normality of outcome variables was assessed using a Shapiro-Wilk statistic. Log transformations were applied prior to analysis for those variables that were not normally distributed.

## Results

**Figure 2:**
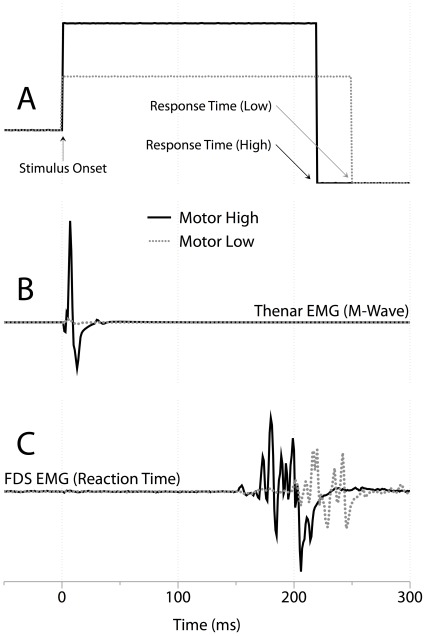
Single trial from a representative subject. (A) Raw stimulus voltage for each of the HIGH intensity (dark black line) and LOW intensity (dashed grey line) stimulus. Upwards deflection indicates stimulus onset and downwards deflection indicates response time for that trial. (B) The resulting raw M-Wave EMG collected from the thenar eminance of the stimulated hand. (C) The raw EMG collected from the flexor digitorum superficialis of the button-pressing limb.

All participants completed all tasks. Note that EEG data from one subject were excluded due to a technical difficulty during data collection. Mean EEG and EMG characteristics for all task conditions are presented in **Table 1**. A sample raw stimulus, M-Wave and EMG response for a characteristic subject are displayed in **Figure 2**. Data presented in the subsequent sections are reported as means ± standard error (SE).

**Table 1:** Characteristics of the EMG & EEG Responses.

	Motor		Sensory	
	High Intensity	Low Intensity	High Intensity	Low Intensity
***EEG***				
**N20 Onset Latency (ms)**	18.9±0.6	19.0±0.6	18.8±0.5	19.1±0.6
**P27 Onset Latency (ms)**	24.2±0.8	24.4±0.8	24.4±0.8	24.4±0.8
**N20–P27 Amplitude (µV)**	4.6±0.7	3.6±0.5	4.2±0.5	3.4±0.6
**PreN Onset (ms, relative to response time)**	−125±7	−135±14	NA	NA
**PreP Onset (ms, relative to response time)**	−30±9	−39±9	NA	NA
**PreN-PreP peak-to-peak Amplitude (µV)**	15.8±1.5	11.9±1.4*	NA	NA
***EMG***				
**M-Wave Onset (ms)**	3.8±0.2	NA	3.9±0.2	NA
**Reaction Time (FDS EMG Onset)(ms)**	170±10	193±11*	NA	NA
**FDS 100 ms Integrated EMG**	NA	81±3% (Of Motor High)*	NA	NA
**Response Time (Mouse Click) (ms)**	241±11	274±15*	NA	NA

* p<0.05.

### Reaction and Response Times

**Figure 3:**
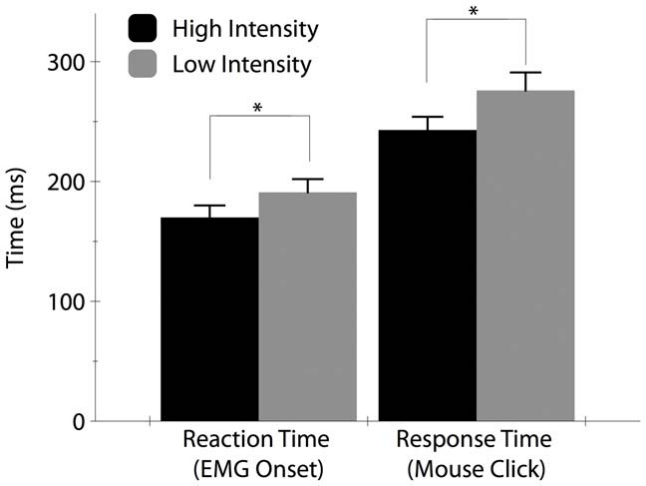
Group averages for reaction and response times by stimulus intensity. The HIGH intensity stimulus (solid black bar) evoked a significantly more rapid reaction and response time than the LOW intensity stimulus (grey bar). (* indicates a significant difference).

The average reaction times and response times in the MOTOR task are shown in **Figure 3**. Overall, reaction times measured from FDS EMG onset were significantly different between stimulus intensities (HIGH = 169.6±10 ms; LOW 192.8±11 ms; F_1,11_ = 33.85, P = 0.0001). Associated response times (time of button press) were also significantly different between stimulus intensities (HIGH = 241±11 ms versus LOW = 274±15 ms; F_1,11_ = 21.68, P = 0.0007). It is noteworthy that the amplitude of the FDS EMG response was also significantly different between HIGH and LOW conditions. The mean normalized 100 ms post-stimulus iEMG for the LOW intensity task was 81±3% of the iEMG following the HIGH intensity stimulus (F_1,11_ = 43.26, P<0.0001). There were significant correlations between the FDS onset latency and mouse click response time in both the LOW and HIGH intensity conditions (r = 0.752, p<0.0001 and r = 0.824, p<0.0001, respectively).

### Electrical Stimulus Intensity

The M-wave onset latencies and amplitudes, measured from the thenar muscle of the stimulated hand, were used to ensure appropriate task-related similarities or differences. M-waves were not evoked following LOW intensity stimuli due to the selected level of stimulus intensity.

The M-wave amplitudes following HIGH intensity stimuli were not significantly different between task conditions (MOTOR: 6.8±0.7 µV; SENSORY: 6.6±0.8 µV; F_1,11_ = 3.82, P = 0.076). Additionally, the onset latency of the M-Wave following the HIGH intensity stimuli, while approaching statistical significance, between task conditions was not meaningfully different (0.1 ms difference; MOTOR: 3.8±0.2 ms; SENSORY: 3.9±0.2 ms; F_1,11_ = 4.22, P = 0.0646).

### SEP Response

**Figure 4:**
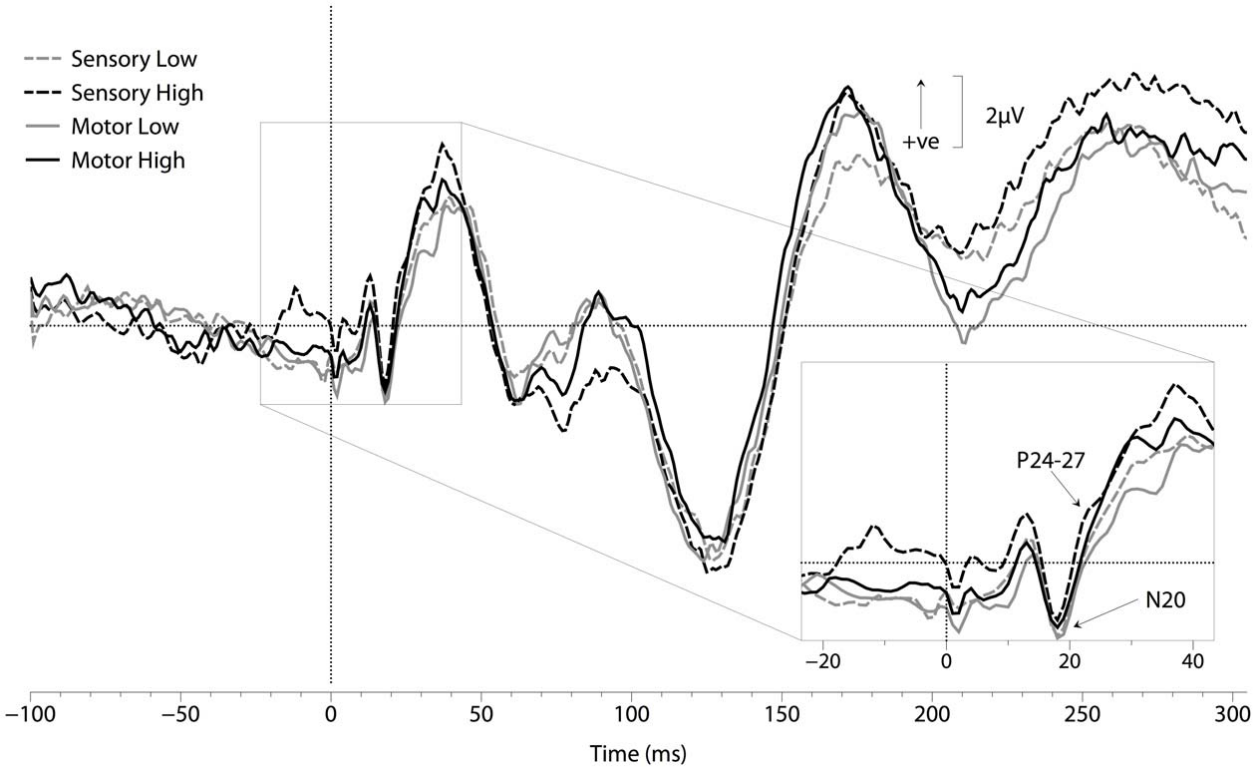
Grand average SEPs. SEPs recorded from the CP4 electrode site (contralateral to stimulated hand) for each of the four task conditions. Stimulus onset is indicated by time ‘0’. SEPs of interest (N20, P24–27) are depicted in the blowout box. There were no significant differences in the latencies and amplitudes of SEPs of interest.

The primary SEP peaks of interest were the N20 and P24–27 (**Figure 4** **and** **Table 1**). These peaks were recorded from an electrode site contralateral to the median nerve stimulus location (CP4). The respective average latencies of the N20 and P24–27 peaks relative to stimulus onset were 19.0±0.6 ms and 24.4±0.8 ms (MOTOR LOW), 18.9±0.6 ms and 24.2±0.8 ms (MOTOR HIGH), 19.1±0.6 ms and 24.4±0.8 ms (SENSORY LOW), 18.8±0.5 ms and 24.4±0.8 ms (SENSORY HIGH). The average N20 to P24–27 peak-to-peak amplitudes for each of the four task conditions were 3.6±0.5 µV (MOTOR LOW), 4.6±0.7 µV (MOTOR HIGH), 3.4±0.6 µV (SENSORY LOW) and 4.2±0.5 µV (SENSORY HIGH). The timing of the peaks for the N20 and P24–27 were not significantly different between the four task conditions (F_3,40_ = 0.23, P = 0.875 and F_3,40_ = 0.17, P = 0.916, respectively). Similarly, the peak-to-peak amplitude difference between the N20 and P24–27 revealed no significant differences across the four task conditions (F_3,40_ = 0.82, P = 0.493).

### ERP Response

**Figure 5:**
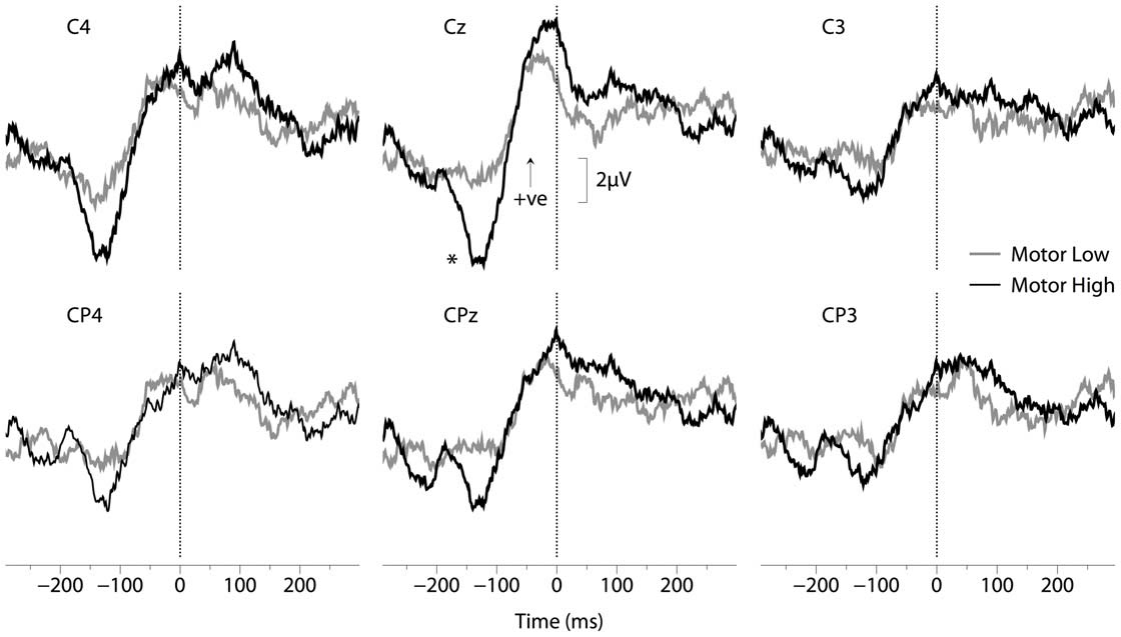
Grand averaged ERPs from six electrode sites. ERPs are averaged relative to response time. The response time is denoted by time = 0. The PreN-PreP amplitude (between 130 ms to 35 ms prior to response time) is significantly greater following the HIGH intensity stimulus (dark black line) compared to the LOW intensity stimulus (grey line) in the Cz electrode.

EEG responses were averaged relative to the response time (mouse click onset) and therefore this analysis was restricted to comparing the LOW and HIGH stimulus for the MOTOR task condition. Overall, there was a consistent large pre-movement negativity (PreN) followed by a positivity evoked just prior to the mouse click (PreP) that was maximal at the Cz cortical site (**Figure 5**). The average PreN-PreP peak-to-peak amplitude was significantly greater following the HIGH intensity stimulus compared to the LOW intensity stimulus (HIGH: 15.8±1.5 µV and LOW: 11.9±1.4 µV; F_1,20_ = 4.48, p = 0.0471). Following the HIGH intensity stimulus, the average latencies of the PreN and PreP responses were −125±7 ms and −30±9 ms, respectively, relative to the response time. Following the LOW intensity stimulus, the average latencies of the PreN and PreP responses were −135±14 ms and −39±9 ms, respectively, relative to response time. PreN and PreP peak latencies were not significantly different between the HIGH and LOW intensity stimuli (F_1,20_ = 0.38, p = 0.545 and F_1,20_ = 0.65, p = 0.428, respectively.)

## Discussion

The primary goal of this study was to explore the relationship of somatosensory stimulus intensity on reaction time, with an emphasis on understanding the electrophysiological correlates associated with stimulus-evoked differences in reaction time. We were most interested in better understanding the determinants of task conditions associated with very rapid reactions times. We relied on SEP and ERP profiles associated with motor and non-motor responses to somatosensory stimuli of varying intensities. This study demonstrated that high intensity somatosensory stimuli evoke more rapid motor responses than low intensity stimuli, consistent with early studies [14], [18]. However, SEP latencies in sites contralateral to the stimulated site did not differ significantly. Additionally, ERPs demonstrated a trend towards a larger cortical negativity approximately 130 ms prior to movement completion during the rapid reactions. We propose that this reveals a link between the reduction in reaction time and a task specific augmentation of motor preparation and execution phases of the transformation.

### Faster Reaction Time Is Not Due To Changes in the Latency of Somatosensory Processing

The finding that reaction time is shorter based on elevated stimulus intensity is not novel. Numerous studies, across various domains, using multiple modalities, have demonstrated the important role of stimulus intensity on an individual's reaction time [15], [19], [29], [30]. However, the attempt to map the mechanism of such a response using EEG following an electrical stimulus is an important and unique step in characterizing the sensorimotor pathways involved in the generation of augmented responses. In the current study, we did not find evidence of a statistically augmented SEP latency or amplitude based on stimulus intensity, despite the significant reduction of reaction time and response latency. Despite the lack of statistical significance surrounding the SEP amplitude, one should not discount the potential biological significance of the 27% increase in SEP amplitude following the high intensity stimulus compared to the low intensity stimulus. This finding reinforces that the high stimulus intensity would have recruited a greater number of afferent axons, resulting in a greater volume of activity at the level of the somatosensory cortex. The complementary lack of change in SEP latency is likely a consequence of the relatively short distance and number of intervening synapses of the somatosensory afferent pathway (to initial cortical SEPs), which limits the capacity to reduce processing speed in this phase. The pathway from the site of stimulation to the contralateral parietal cortex, where the evoked potential was recorded, contains few synapses (located at the gracile nucleus in the medulla, the ventral posterior lateral nucleus in the cerebral cortex and terminating at the somatic sensory cortex) therefore limiting the ability to significantly reduce the N20 and P24–27 latencies. Previous evidence has demonstrated that more rapid reaction times could be generated when a stimulus can be sufficiently anticipated, by elevating the baseline level of activity integrator neurons, which initiates the response cascade [31]. However, given the absence of differences in the N20 and P24–27 latencies between stimulus intensities, it is unlikely that participants were able to anticipate the impending stimulus in the present study. Notably, the similarity between the N20 and P24–27 waveforms and latencies indicates that the reduction of reaction time must occur further along the sensorimotor pathway at the level of cortical integration or during the efferent conduction to the flexor digitorum muscle.

### Motor Response Following a Stimulus Does Not Alter the Latency or Amplitude of the Early SEPs

This study also revealed no significant differences between the latencies or amplitudes of the N20 and P24–27 dependent on whether the individual were to generate a motor response or sit quietly following a stimulus, regardless of stimulus intensity. Because the tasks (MOTOR/SENSORY) were delivered in blocks, participants had knowledge of the response following the stimulus. This further indicates that the immediate EEG events following a stimulus are not sensitive to variations in the task demands and that the afferent pathway is largely stereotyped with respect to its conduction latency and amplitude of activation. The N20 is generally associated with activity in area 3b at the postcentral gyrus and has been termed an exogenous component of the SEP cascade, indicating that its latency does not vary based on the cognitive task associated with the stimulus [32]. The current study builds upon the previous knowledge by indicating that the requirement to generate movement following a discrete stimulus has no effect on the somatosensory processing latency of the stimulus.

Preparation for a motor task is commonly identified with a pre-movement cortical negativity, known as the Bereitschaftspotential, in frontal planning areas, such as the supplementary motor area (SMA) [33]. However, pre-stimulus motor preparation was not present in the current study, since temporal variability in stimulus delivery removes or attenuates the Bereitschaftspotential [34]. This provides further evidence for the hypothesis that, when stimuli are temporally unpredictable, the latency of events along the afferent pathway to the contralateral somatosensory cortex is not susceptible to deviations based on stimulus properties or task conditions.

### The Amplitude of ERPs Appear to be Related to the Stimulus Intensity and Motor Tuning

There was significantly greater PreN-PreP peak-to-peak amplitude following the higher intensity stimulus. Given the variability in response time, both within and between subjects, the presence of a predominant residual negativity to positivity in the Cz EEG site is an important finding. The onset of FDS EMG activity occurred approximately 73 ms and 85 ms in advance of the mouse click following the HIGH and LOW intensity stimuli, respectively. Importantly, the average peak time of the PreN potential occurs prior to the onset of EMG activity (125 ms prior to mouse click for HIGH, 135 ms for LOW), whereas the PreP potential occurs after the EMG onset, but prior to the mouse click (29 ms prior to mouse click for HIGH, 29 ms prior to mouse click for LOW). The varying amplitude of the PreN-PreP based on stimulus intensity could signify an important variation of the transformation from somatosensory areas to pre-motor areas such as SMA and primary motor cortex that acts to encode and plan the appropriate response, despite the intensity of the stimulus having no effect on early SEPs such as N20 and P24–27. It is conceivable that the PreN-PreP amplitude influences the processing time (reaction time) itself or the amplitude of muscle activity required for a given task. Though not statistically significant, **Figure 5** demonstrates a lateralization of the PreN-Pre-P amplitude towards cortical sites contralateral to the stimulated limb following a high intensity stimulus. This may indicate a relationship between the translation of somatosensory stimulus information, such as the elevated stimulus intensity, to motor cortical areas, such as SMA. We observed greater integrated EMG activation within 100 ms post-EMG onset following the HIGH stimulus compared to the LOW stimulus, as has been demonstrated previously [35]. This finding raises important links between the CNS mechanism that results in increased motor response amplitude and this associated with a reduction of reaction time latencies.

Saccadic movements are often used in the study of reaction time mechanisms due to its well-mapped pathways. Bell et al. [19] measured the onset of neuronal activity in the superior colliculus of monkey and noted that activity occurred earlier following a high intensity stimulus compared to a low intensity stimulus, which resulted in reduced saccade latency. How is the latency of processing reduced in these situations? Allocation of attention is frequently cited as a potential modulator of reaction time latency [36], [37], [38]. The effect of attention on reaction time is often extracted by examining the variability of the reaction time within subjects. Previous studies have demonstrated that higher intensity stimuli elicit a response with lower intra-subject variation, and this finding is often attributed to an increased capacity to allocate attention to the stimulus [36]. Processing and comprehension of stimulus properties as well as the directing of attentional resources towards the stimuli may have an important relationship with the elevated cortical negativity over the frontal central cortical observed in the current study, although further investigation is required to confirm this observation.

### Conclusions

The current study set out to investigate the electrophysiological determinants (SEPs and ERPs) of rapid reaction times evoked by differences in stimulus intensity. We demonstrated that elevated stimulus intensity does not have a statistically significant effect on the latency and amplitude of SEPs, while generating significantly shorter reaction and response times. Additionally, ERPs relative to the response time demonstrated a significantly greater pre-movement negativity to positivity following the high intensity stimulus, which may be related to movement planning and evocation of more rapid responses. This work has important implications for understanding the mechanisms by which the CNS processes the various characteristics of discrete stimuli that could be used to assist in novel rehabilitation methods for individuals who are characteristically slow to respond, such as individuals who have suffered a stroke. Further work is required to explore the potential role that emotional and attentional cortical centers may play to mediate the latency of responses when rapid responses are required.
