# Correction: Electrophysiological Correlates of Changes in Reaction Time Based on Stimulus Intensity

**DOI:** 10.1371/annotation/b48d6298-409f-45a9-8e4c-32eef9dbd188

**Published:** 2014-01-03

**Authors:** Bimal Lakhani, Albert H. Vette, Avril Mansfield, Veronica Miyasike-daSilva, William E. McIlroy

There was a statistical error that affects the "SEP Response" and "ERP Response" sections of the Results, the "Faster Reaction Time Is Not Due To Changes in the Latency of Somatosensory Processing" section of the Discussion, and the Conclusions. The corrected text can be found here: http://www.plosone.org/corrections/pone.0036407.001.cn.docx

